# Stroke and thromboembolic event rates in atrial fibrillation according to different guideline treatment thresholds: A nationwide cohort study

**DOI:** 10.1038/srep27410

**Published:** 2016-06-06

**Authors:** Peter Brønnum Nielsen, Torben Bjerregaard Larsen, Flemming Skjøth, Thure Filskov Overvad, Gregory Y. H. Lip

**Affiliations:** 1 Aalborg Thrombosis Research Unit, Department of Clinical Medicine, Faculty of Health, Aalborg University, Aalborg, Denmark; 2 Department of Cardiology, Atrial Fibrillation Study group, Aalborg University Hospital, Aalborg, Denmark; 3 Unit of Clinical Biostatistics and Bioinformatics, Aalborg University Hospital, Aalborg, Denmark; 4 University of Birmingham Institute for Cardiovascular Sciences, City Hospital, Birmingham, United Kingdom

## Abstract

Contemporary guidelines suggest anticoagulant treatment decisions in atrial fibrillation (AF) patients to be based on risk stratification for stroke. However, guidelines do not agree on the threshold for treatment initiation. We explored the variation in thromboembolic event rates in a non-anticoagulated AF population, according to different guideline threshold and methodological approaches. AF patients between 1998 and 2014 free from anticoagulant treatment were identified. Event rates for ischemic stroke and ischemic stroke/systemic embolism were explored. The overall ischemic stroke rate was 3.20 per 100 person-years (‘formal rate assessment’). For patients with a CHA_2_DS_2_-VASc score of 1 the ischemic stroke rate was 0.97 when using a ‘formal rate assessment’, 0.62 when using a ‘conditioning on the future’ approach, and 0.93 when using a ‘censoring approach’. Rates for thromboembolism for the ‘European treatment threshold’ (CHA_2_DS_2_-VASc score of 1, males only) ranged 1.17 to 1.53. Rates for the ‘U.S. treatment threshold’ (CHA_2_DS_2_-VASc of 2) ranged from 1.95 to 2.33. Thromboembolic event rates differed markedly in non-anticoagulated AF patients according to the conflicting European and U.S. guideline treatment thresholds. Second, the choice of methodological approach has implications, thus we recommend using the censoring approach for event rate estimation among AF patients not on treatment.

Atrial fibrillation (AF) is the most common sustained cardiac arrhythmia and with the aging of populations the prevalence of AF is expected to increase^1^. AF is estimated to affect up 12 million people in the United States and more than 17 million in Europe within the next 40 to 50 years^2^,^3^. AF is recognized as an important healthcare burden^4^ given the associations with stroke^5^, heart failure^6^, and mortality^7^,^8^. The risk of stroke and mortality can be effectively reduced by use of oral anticoagulant treatment^9^. However, use of anticoagulants also increases the risk of bleeding, why the decision of stroke prophylactic treatment should balance the harms against the benefits from treatment.

Contemporary guidelines from the European Society of Cardiology, the American College of Cardiology, the American Heart Associations, and the Heart Rhythm Society for managing stroke prophylactic treatment in AF patients advocate for the use of the CHA_2_DS_2_-VASc score (Congestive heart failure[1 point], Hypertension[1 point], Age 75 years[2 points], Diabetes[1 point], Stroke[2 points], Vascular disease[1 point], Age 65–74 years [1 point], and female sex category[1 point]) for stroke risk stratification^10^,^11^,^12^. In ‘low risk’ patients with no additional stroke risk factors, oral anticoagulant treatment is not recommended. Oral anticoagulants are clearly recommended for high risk patients with two or more known risk factors (a score of ≥2). However, the European and the U.S. guidelines do not agree on the threshold for treatment recommendation. The U.S. guideline offers a Class IIb recommendation on either no treatment, aspirin therapy, or oral anticoagulant treatment in AF patients with a CHA_2_DS_2_-VASc score of 1^11^. The European guideline recommends that female sex as a single risk factor should be disregarded, and hence, low risk is defined as a CHA_2_DS_2_-VASc score of 0 in males, 1 in females, where no antithrombotic therapy is recommended. Thus, oral anticoagulant treatment should be considered in AF patients with a one stroke risk factor (i.e. CHA_2_DS_2_-VASc score of 1 in males, or 2 for females; Class IIa recommendation), or recommended in those with a CHA_2_DS_2_-VASc score ≥2 (Class I recommendation)^12^. The discrepancy between guideline recommendations on treatment threshold is shown in Table 1.

Guideline writing committees have based their recommendations on data from the Danish nationwide registries^13^,^14^. Lately, the reported Danish event rates of thromboembolism have been questioned^15^, but event rates clearly differ by populations studied (e.g. community vs hospitalized cohorts) and study methodology. Indeed, Friberg *et al.* suggested that oral anticoagulant treatment was unlikely to benefit AF patients with a CHA_2_DS_2_-VASc score of 1 based on data from the nationwide Swedish cohort^16^. Methodological concerns on this study leading to biased rates have been raised^17^.

In this study, we aimed to investigate the thromboembolic event rates in a non-anticoagulated atrial fibrillation population, in relation to different guideline cut-off values. Second, we studied the impact of different methodological approaches when investigating event rates in an untreated population.

## Methods

This was an observational study of a nationwide population of unselected hospitalized nonvalvular AF patients identified from and followed in Danish administrative registries.

### Study population and data sources

Study data was obtained by cross-linkage of three nationwide databases: (I) the Danish National Prescription Registry, which records purchase date, Anatomical Therapeutic Chemical [ATC] classification code, and package size for every prescription purchase in Denmark since^18^; (II) the Danish National Patient Register which contains discharge diagnoses for all hospital admissions in Denmark defined in terms of International Classification of Diseases revision 10 (ICD-10) since 1994^19^; and (III) the Danish Civil Registration System which holds information on sex, date of birth, vital and emigration status^20^. Ethical approval is not required for register-based studies in Denmark.

We identified first-time hospitalized AF (ICD-10: I48) patients from January 1^st^ 1998 through August 30^th^ 2014. We restricted the cohort by excluding those with expected valvular AF (patients with mitral stenosis [ICD-10: I05] or valve replacement [ICD-10: Z952 Z953 Z954]); patients who immigrated within two years prior to their AF diagnosis were also excluded. Finally we excluded those who had purchased anticoagulants within one year prior to the AF diagnosis. Past-year oral anticoagulant prescriptions claims were identified from the ATC code sections on “antithrombotic agents” (B01AA, B01AE, B01AF). The final study population comprised nonvalvular AF patients who were not treated with oral anticoagulants.

### Comorbidities and outcomes

Information on comorbidities and concomitant medication was obtained from the registries at the date of hospital discharge in relation to AF diagnosis (index date). History of cardiovascular comorbidities was ascertained in terms of records according to the ICD-10 codes in the Danish National Patient Registry. We calculated the CHA_2_DS_2_-VASc score as previously described^21^,^22^. Specifically, we identified previously recorded diagnoses and concomitant medication, which were utilized to calculate an individual risk score, see Table S1 for detailed information on definition for each risk component. Importantly, diagnoses acquired during hospitalization for AF was also included in the assessment of baseline comorbidities.

Patients were followed in the Danish National Patient Registry from the index date and up to five years until the occurrence of an outcome of interest, death, emigration, or end of study, whichever came first. The endpoints of interest were ischemic stroke (ICD-10: I63) and the composite endpoint of ischemic stroke and systemic embolism (ICD-10: I63; I74).

For quantifying thromboembolic risk we constructed four different risk categories defined according to points on the CHA_2_DS_2_-VASc score: ‘truly low risk’ [CHA_2_DS_2_-VASc = 0 for males and 1 for females]; ‘European treatment threshold’ [CHA_2_DS_2_-VASc = 1, males]; ‘U.S. treatment threshold’ [CHA_2_DS_2_-VASc = 2]; and ‘high risk’ [CHA_2_DS_2_-VASc >2]. It should be noted that a consequence from these definitions, female patients with a CHA_2_DS_2_-VASc = 2 (i.e. one additional risk factor than female sex) would be included in the ‘U.S. treatment threshold’, albeit this categorization would also trigger a treatment recommendation according to the European Society of Cardiology guideline (CHA_2_DS_2_-VASc score of 1 in males, or 2 for females).

### Statistical analysis and study design

Baseline characteristics were summarized descriptively. We calculated crude event rates for all endpoints and risk strata as events per 100 years at risk. Additionally, we ascertained the thromboembolic event rates for each point on the CHA_2_DS_2_-VASc score scale. To visualize how the risk evolved over time since index date, we calculated and displayed the one-year cumulative incidence of ischemic stroke taking into account the competing risk of death in each strata given by the risk categories defined above^23^. We performed analyses with STATA version 14 (StataCorp, TX, U.S).

As the rate and risk estimates will depend on how patients are included in the study, we evaluated three different methodological approaches to patient inclusion and restriction of patient follow-up: (1) Include all patients from baseline and ignore that patients may initiate treatment with oral anticoagulant treatment during follow-up [‘formal rate assessment’]. This approach would likely underestimate the ‘real’ event rates as this treatment effectively reduces the risk of thromboembolism compared to no treatment^9^. (2) A ‘conditioning on the future’^24^ approach by excluding all patients at baseline that initiates oral anticoagulant treatment during follow-up [ATC codes: B01AA; B01AE07; B01AF01; B01AF02]. This approach corresponds to that of Friberg *et al.*^16^, and might theoretically underestimate the event rates, since the excluded patients are unlikely to be a random sample of the source population of interest, e.g., oral anticoagulant treatment is often initiated in patients surviving an ischemic event. (3) A censoring approach by right-censoring observation time when a prescription of an oral anticoagulant treatment is claimed^25^. This approach allows for inclusion of observation time and thromboembolic events before a treatment initiation decision.

We conducted sensitivity analyses to investigate the robustness of the obtained results from the main analyses. As the type of hospital diagnosis (primary or secondary diagnosis) might affect thromboembolic event rates^16^, we conducted sensitivity analyses by restricting outcomes to only primary diagnosed events. We also investigated if any temporary effect in data was present by restricting the cohort to include data only from the five previous years (2009–2014). Lastly, although aspirin does not provide optimal stroke prophylaxis in AF patients, some evidence of benefit in terms of lower stroke risk compared to no treatment may still persist^9^. Hence, we analyzed if exclusion of patients with baseline aspirin treatment (ATC code: B01AC06) would impact the event rates by underestimating the ‘natural’ thromboembolic event rate in an untreated AF population.

## Results

During the study period, a total of 238,270 patients received a first-time hospital diagnosis of nonvalvular AF, while 1,394 of these patients immigrated within two years prior to their AF diagnosis and were not included. After exclusion of patients with baseline anticoagulant treatment (n = 38,179), a total of 198,697 individual patients initially free from oral anticoagulant treatment contributed to the analyses. The mean age was 75 years and 49% were females; the mean CHA_2_DS_2_-VASc score was 2.9 [Table 2]. A total of 15% was categorized as being ‘truly low risk’; 9% had indication for treatment according to the ‘European treatment threshold’; 18% according to the ‘U.S. treatment threshold’; while 58% were at ‘high risk’ of thromboembolic events.

Thromboembolic events were observed according to the defined stroke risk strata, with 688 events in ‘truly low risk’ patients, 776 events in ‘European treatment threshold’ patients, 2,133 in ‘U.S. treatment threshold’ patients, and 11,937 in ‘high risk’ patients. Of these, 93% were ischemic strokes and 7% were systemic embolism.

We observed an overall ischemic stroke rate per 100 person-years of 3.20 and for the composite endpoint of ischemic stroke/systemic embolism of 3.42 (using a formal rate assessment approach) during a mean follow-up time of 2.9 years. During follow-up 99,355 patients initiated treatment with oral anticoagulants. Thus, when imposing the ‘conditioning on the future’ approach, the number of patients investigated was reduced to 99,342 patients.

**Figure 1:**
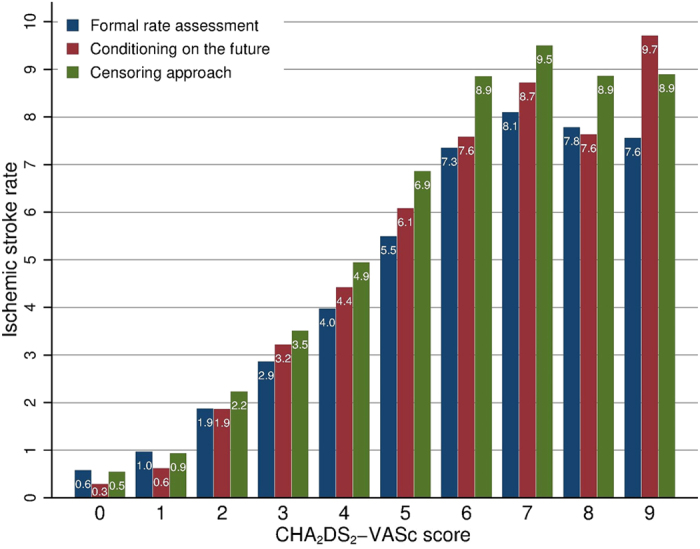
Ischemic stroke rates. Ischemic stroke rates according to different levels of baseline CHA_2_DS_2_-VASc score stratified by methodological approach.

Figure 1 shows the event rates of the primary outcome corresponding to points on the CHA_2_DS_2_-VASc score and stratified according to study design [see Table S2 for tabulated information].

The three different methodological approaches entailed different event rates at different risk levels. The event rate of ischemic stroke for patients with a CHA_2_DS_2_-VASc score of 1 was 0.97 when using ‘formal rate assessment’, while this rate markedly decreased to 0.62 when applying a ‘conditioning on the future’ approach. In general, the ‘censoring approach’ resulted in highest event rates for patients at high risk of stroke (i.e. CHA_2_DS_2_-VASc score >2) compared to the two other methodological approaches.

Assessing the thromboembolic event rate (composite endpoint of ischemic stroke and systemic embolism) at different risk thresholds revealed that ‘truly low risk’ patients had an event rate that ranged from 0.30 to 0.60 dependent on study design [Table 3]. For males with a CHA_2_DS_2_-VASc score of 1 (‘European treatment threshold’), the highest event rate (1.53) was observed when person-time was censored at the date of oral anticoagulant treatment initiation (‘censoring approach’), while the lowest observed rate was 1.17 using the conditioning on the future approach. For the ‘U.S. treatment threshold’ (CHA_2_DS_2_-VASc of 2) the formal event rate was 1.95, rising to 2.33 with the censoring approach. Patients categorized as ‘high risk’ generally had a thromboembolic event rate above 4, with the highest rate (5.49) using the censoring approach.

**Figure 2:**
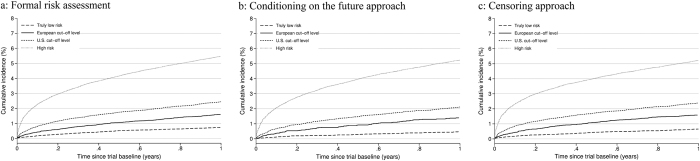
(**a–c**) Cumulative risk of ischemic stroke for different risk definitions and methodological approaches. (**a**) Formal risk assessment (**b**) Conditioning on the future approach. (**c**) Censoring approach.

The cumulative incidence (risk) of ischemic stroke during the first year after index date is depicted in Fig. 2a–c for each of the study designs. The risk was above 1% for all definitions of ischemic stroke risk apart from ‘truly low risk’ category.

### Sensitivity analyses

Sensitivity analyses investigating the thromboembolic event rates using only primary diagnosed events slightly attenuated the observed rates [Table S3–4]. The formal rate assessment of thromboembolism displayed a rate of 1.06 for the ‘European treatment threshold’ and 1.57 for the ‘U.S. treatment threshold’. Restricting the cohort by using data from five previous years (2009–2014) did not affect the event rates [Table S5]. Investigating a cohort restricted to patients with no baseline aspirin treatment did not materially affect the results [data not shown].

## Discussion

This nationwide observational study of AF patients free from oral anticoagulant treatment provides important insights into thromboembolic event rates and the consequences of guideline treatment thresholds and different methodological approaches. We found that thromboembolic event rates varied according to guidelines risk strata, and most importantly, according to study design.

The thromboembolic event rates per 100 person-years for the ‘European treatment threshold’ and the ‘U.S. treatment threshold’ were 1.31 and 1.97, respectively. Imposing an inappropriate methodological approach (i.e. conditioning on the future) resulted in decreased thromboembolic event rates for patients with a CHA_2_DS_2_-VASc score ranging 0 to 2. Specifically, this approach had an important impact (in comparison to the other study designs) for patients with a CHA_2_DS_2_-VASc score  =  1. Constructing a non-treated AF population by right-censoring person-time at the date of anticoagulant treatment initiation yielded the highest thromboembolic event rates in each risk strata (disregarding the ‘truly low risk’ strata). It is our belief that this approach is the appropriate way to obtain event rate estimates for a non-treated population.

One explanation of the observation of deflated event rates when using the ‘conditioning on the future’ approach could be related to prescriber behavior. It could be hypothesized that patient and/or physician preferences for treatment in seemingly “low risk” AF patients have been not to consider anticoagulant treatment. However, such a patient might subsequently have encountered an ischemic stroke promptly triggering a prescription of an anticoagulant agent (assuming that the patient survives). In the ‘conditioning on the future’ approach such a hypothetical patient was not contributing person-time or events. Therefore, we warrant caution in interpretation of the event rates provide by Friberg *et al.* suggesting that patients with a CHA_2_DS_2_-VASc score of 1 (rate of 0.5 to 0.9 per 100 person-years) might not benefit from oral anticoagulant treatment^16^.

Nevertheless, overall event rates of stroke and thromboembolism may vary between different AF populations as a consequence of study settings, origin of population, or health care plan or not^26^. Indeed, a hospitalized AF cohort may represent a population with more severe conditions compared to that of an outpatient cohort with AF patients enrolled by a group-model health organization with follow-up status dependent upon a healthcare plan^27^. This was reflected directly onto thromboembolic event rates in the Anticoagulant and Risk Factors in Atrial Fibrillation (ATRIA) cohort that reported an annualized rate of 2.1%^28^. In contrast, data from the U.S. based National Registry of Atrial Fibrillation (that included hospitalized AF patients) reported a stroke rate of 4.4 per 100 person-years^29^. Importantly, considerable differences in event rates according to CHA_2_DS_2_-VASc score has also been observed^26^, which may pertain in part to the simplicity and reductionist formulation of the CHA_2_DS_2_-VASc score (with a view to practicality) and its incapacity to take disease severity into account. In the Danish study by Olesen *et al.* the primary endpoint was a composite of ischemic stroke, systemic embolism, and pulmonary embolism^14^. Studying a composite endpoint will inevitably result in a higher event rate compared to studying a lone endpoint. Thus, it is pivotal to construct study endpoints in accordance with the research question in mind in order to gain appropriate insight into clinical practice.

Ultimately, decision-making would need to consider the net clinical benefit of thromboprophylaxis by considering stroke reduction with oral anticoagulant treatment, versus the potential for harm due to serious bleeding (especially intracranial haemorrhage). In a comprehensive net clinical benefit analysis, we previously found that with a single stroke risk factor (i.e. CHA_2_DS_2_-VASc score 1 in males, and 2 in females), the net clinical benefit was positive for warfarin vs untreated, and for warfarin vs aspirin^30^. Using a non-vitamin K antagonist oral anticoagulant (NOAC), the net clinical benefit is anticipated to be even greater^31^. In decision analysis model, Eckman *et al.* found the tipping point threshold for initiating warfarin was a stroke rate of 1.7%/year, but for NOACs (specifically, dabigatran), the treatment threshold was a stroke rate of 0.9%/year^32^. Nonetheless, this approach is limited by the lack of clinical trials specifically addressing only those patients with a single stroke risk factor based on the CHA_2_DS_2_-VASc score, although recent phase 3 randomized trials with dabigatran and apixaban did include a few of such patients^33^.

Given that clinical scores only have modest predictive value in terms of discriminatory ability, the CHA_2_DS_2_-VASc score is particularly useful for identifying low risk patients with ischemic stroke rates <1%/year^10^, the approach should be to initially identify low risk patients (i.e. CHA_2_DS_2_-VASc score 0 in males, and 1 in females) who do not need any antithrombotic therapy. The subsequent step would be to offer stroke prevention to those with 1 or more stroke risk factors. Indeed, stroke risk scores like CHA_2_DS_2_-VASc score are meant to balance simplicity and practicality against identifying those ‘at risk’, without the need for complex weighted formulae, often derived from multivariate analyses of highly selected trial cohorts. Indeed, it may not be possible, or even necessary for risk scores to accurately predict the exact absolute risk across populations. A risk prediction model may serve well as a clinical decision-tool if it provides reliable thresholds at which important dichotomous clinical decisions can be made; for example, use of anticoagulation vs no anticoagulation. This stepwise approach of the European Society of Cardiology and N(ational Institute for Health and Care Excellence guidelines has been validated showing guideline adherent therapy is associated with improved outcomes^34^,^35^.

We identified AF patients based on database records in the registry. Although this diagnosis has been shown to be highly accurate^36^, we only included hospitalized AF patients. Thus our results may not generalize into AF patients identified in general practice, who might have a lower risk of thromboembolism compared to hospitalized AF patients. However, we are not dealing with a patient population that has a static risk profile, given the elderly age, multiple comorbidities, and frequent hospitalizations associated with AF. In general, we cannot rule out the risk of misclassification and ascertainment error. The ischemic stroke outcome has been previously validated in the Danish National Patient Registry with a positive predictive value of more than 97%^37^. Nevertheless, the vast majority of the studied outcomes were ‘lone’ ischemic strokes. In general, the positive predictive value of the coding in the Danish National Patient Register has been shown to be consistently high. Although we have shown that event rates vary depending on study design, none of the reported rates are likely to accurately reflect the ‘real’ event rate. For example, in case of lack of coding for a specific disease included as a risk component in the CHA_2_DS_2_-VASc score, this could misclassify patients into an incorrect risk strata, that is, some patients with CHA_2_DS_2_-VASc = 1 in our study may in reality have a score of 2 due to a diagnosis of hypertension that has escaped our definition. This would theoretically overestimate the event rates associated with the lower risk categories. However, some factors would also contribute to an underestimation of the ‘real’ rates. For example, death due to undiagnosed stroke would not be coded; in Denmark, very few deceased people have an autopsy performed, just as some ischemic strokes may be missed if coded as unspecified stroke – a diagnosis we did not include in our outcome definition. Finally, it is important to emphasize that our estimates of thromboembolic rates do not equal absolute risks of stroke or thromboembolism. It is essential to make this distinction between risks and rates especially when a risk of competing events (such as death) is present.

### In conclusion

We observed thromboembolic event rates in a non-anticoagulated atrial fibrillation population, related to different guideline cut-off values, i.e. up to 1.53 per 100 person-years in untreated atrial fibrillation patients according to the European treatment threshold, and up to 2.33 for the U.S. treatment threshold. Second, different methodological approaches when investigating event rates in an untreated population have implications for the reported event rates.

## Additional Information

**How to cite this article**: Nielsen, P. B. *et al.* Stroke and thromboembolic event rates in atrial fibrillation according to different guideline treatment thresholds: A nationwide cohort study. *Sci. Rep.* **6**, 27410; doi: 10.1038/srep27410 (2016).

## Supplementary Material

Supplementary Information

## Figures and Tables

**Table 1:** Overview of guideline recommendations on antithrombotic therapy according to different levels of CHA_2_DS_2_-VASc score.

	U.S. guideline recommendations^†^		European guideline recommendations^‡^	
	Women	Men	Women	Men
CHA_2_DS_2_-VASc = 0	N/A^*^	No therapy	N/A^*^	No therapy
CHA_2_DS_2_-VASc = 1	Oral anticoagulation, aspirin, or no therapy		No therapy	Oral anticoagulation
CHA_2_DS_2_-VASc ≥2	Oral anticoagulation		Oral anticoagulation	

*Women cannot score 0, as female sex triggers 1 point in the CHA_2_DS_2_-VASc score.

^†^The American College of Cardiology, the American Heart Associations, and the Heart Rhythm Society (ACC/AHA/HRS). ^‡^European Society of Cardiology (ESC). N/A: Not available.

**Table 2:** Patient baseline characteristics stratified according to stroke risk definitions.

Clinical characteristics	Total population	Truly low risk	European treatment threshold	U.S. treatment threshold	High risk
Number of patients	198,697	29,174 (15%)	17,592 (9%)	35,996 (18%)	115,935 (58%)
Age, mean (IQR)	75 (65–83)	55 (47–61)	66 (60–70)	71 (65–78)	81 (75–86)
64–74 years	148,480 (75)	0 (0)	9,952 (57)	27,731 (77)	110,797 (96)
≥75 years	98,998 (50)	0 (0)	0 (0)	11,763 (33)	87,235 (75)
Female sex	96,529 (49)	10,120 (35)	0 (0)	12,280 (34)	74,129 (64)
Congestive heart failure*	34,817 (18)	0 (0)	1,491 (8)	2,819 (8)	30,507 (26)
Heart failure	20,413 (10)	0 (0)	294 (2)	1,068 (3)	19,051 (16)
Left ventricular dysfunction	30,833 (16)	0 (0)	1,409 (8)	2,575 (7)	26,849 (23)
Hypertension	74,113 (37)	0 (0)	3,972 (23)	9,337 (26)	60,804 (52)
Diabetes	23,785 (12)	0 (0)	960 (5)	2,530 (7)	20,295 (18)
Prior thromboembolism*	32,382 (16)	0 (0)	0 (0)	1,086 (3)	31,296 (27)
Ischemic stroke	24,230 (12)	0 (0)	0 (0)	793 (2)	23,437 (20)
Systemic embolism	1,615 (1)	0 (0)	0 (0)	31 (0)	1,584 (1)
Transient ischemic attack	10,198 (5)	0 (0)	0 (0)	354 (1)	9,844 (8)
Vascular disease*	37,045 (19)	0 (0)	1,217 (7)	3,360 (9)	32,468 (28)
Myocardial infarction	25,768 (13)	0 (0)	938 (5)	2,469 (7)	22,361 (19)
Aortic plaque	353 (0)	0 (0)	7 (0)	28 (0)	318 (0)
Peripheral vascular disease	15,153 (8)	0 (0)	320 (2)	1,098 (3)	13,735 (12)
Mean CHA_2_DS_2_-VASc score (SD)	2.9 (1.8)	0.4 (0.5)†	1 (0)	2 (0)	4.1 (1.2)

Data are n (%) unless indicated otherwise.

*Patients can have had one or more comorbidities from each entity.

^†^Contribution from female sex category.

IQR: Interquartile range.

SD: Standard deviation.

**Table 3:** Thromboembolic event rates in relation to different methodological approaches and stratified according to cut-off values of stroke risk based on the CHA_2_DS_2_-VASc score.

Risk stratification	CHA_2_DS_2_-VASc score	Formal rate assessment			Conditioning on the future approach			Censoring observation at oral anticoagulant treatment		
		Events	Person-years	Rate/100 person-years	Events	Person-years	Rate/100 person-years	Events	Person-years	Rate/100 person-years
Truly low risk	0 (1 for females)	688	114,504	0.60	168	56,053	0.30	400	73,873	0.54
European treatment threshold	1 (males)	812	61,773	1.31	200	17,067	1.17	402	26,324	1.53
U.S. treatment threshold	2	2,245s	114,034	1.97	792	40,576	1.95	1,305	55,920	2.33
High risk	>2	12,737	288,944	4.41	6375	129,572	4.92	8,569	156,032	5.49
