# Supplementary material for: Stroke and thromboembolic event rates in atrial fibrillation according to different guideline treatment thresholds: A nationwide cohort study
Source: Sci Rep. 2016 Jun 6;6:27410. doi: 10.1038/srep27410 (PMC4893655; doi:10.1038/srep27410)
Supplement: Supplementary Information [file srep27410-s1.pdf]

Stroke and thromboembolic event rates in atrial fibrillation according to different guideline treatment thresholds: A nationwide study

Peter Brønnum Nielsen, PhD

Torben Bjerregaard Larsen, PhD

Flemming Skjøth, PhD

Thure Filskov Overvad, MD

Gregory Y.H. Lip, MD

Supplemental information

List of contents:

eTable 1: Definitions on comorbidity and concomitant medication according to ICD-10 codes and ATC-codes.

eTable 2: Thromboembolic event rates according to different levels of baseline CHA_2DS_2-VASc score stratified by methodological approach.

eTable 3: Primary diagnosed thromboembolic event rates according to different levels of baseline CHA_2DS_2-VASc score stratified by methodological approach.

eTable 4: Primary diagnosed thromboembolic event rates in relation to different methodological approaches stratified according to cut-off values of stroke risk based on the CHA_2DS_2-VASc score.

eTable 5: Thromboembolic event rates according to different levels of baseline CHA_2DS_2-VASc score using data from previous five years (2009 – 2014).

eTable 1: Definitions on comorbidity and concomitant medication according to ICD-10 codes and ATC-codes. Conditions marked with † was used in the calculation of the CHA_2DS_2-VASc score.

	International Classification of Diseases 10th revision (ICD-10) code	Anatomical Therapeutic Chemical (ATC) code
Condition		
†Congestive heart failure	I11.0; I13.0; I13.2; I42.0; I50	CO3C
†Left ventricular dysfunction	I50.1; I50.9	
†Hypertension		See specified definition*
†Diabetes mellitus	E10.0; E10.1; E10.9; E11.0; E11.1; E11.9	A10
†Ischemic stroke	I63	
†Systemic embolism	I74	
†Transient ischemic disease	G45	
†Aortic plaque	I70.0	
†Peripheral arterial disease	I70.2-I70.9; I71; I73.9; I74	
†Myocardial infarction	I21-I23	
Atrial fibrillation	I48	
**Medication**		
Dabigatran		B01AE07
Rivaroxaban		B01AE07

Apixaban	B01AF02
Warfarin	B01AA03
Aspirin	B01AC06
Beta-blockers	C07
Alpha adrenergic blockers	C02A, C02B, C02C
Non-loop diuretics	C02DA, C02L, C03A, C03B, C03D, C03E, C03X, C07C, C07D, C08G, C09BA, C09DA, C09XA52
Vasodilators	C02DB, C02DD, C02DG, C04, C05
Calcium channel blockers	C07F, C08, C09BB, C09DB
Renin-angiotensin system inhibitors	C09

* We identified subjects with hypertension† from combination treatment with at least two of the following classes of antihypertensive drugs:

I. Alpha adrenergic blockers (C02A, C02B, C02C)

II. Non-loop diuretics (C02DA, C02L, C03A, C03B, C03D, C03E, C03X, C07C, C07D, C08G, C09BA, C09DA, C09XA52)

III. Vasodilators (C02DB, C02DD, C02DG, C04, C05)

IV. Beta blockers (C07)

V. Calcium channel blockers (C07F, C08, C09BB, C09DB)

VI. Renin-angiotensin system inhibitors (C09).

eTable 2: Thromboembolic event rates according to different levels of baseline CHA_2DS_2-VASc score stratified by methodological approach.

	**Formal rate assessment**					
	Ischemic stroke			Ischemic stroke/systemic embolism		
CHA_2DS_2-VASc score	Events	Person-years	Rate/100 person-years	Events	Person-years	Rate/100 person-years
0	432	74,945	0.58	469	74,856	0.63
1	982	101,514	0.97	1,031	101,421	1.02
2	2,133	114,216	1.87	2,245	114,034	1.97
3	3,516	123,089	2.86	3,730	122,742	3.04
4	3,582	90,296	3.97	3,803	89,955	4.23
5	2,569	46,783	5.49	2,753	46,479	5.92
6	1,574	21,402	7.35	1,698	21,202	8.01
7	566	6,988	8.10	611	6,914	8.84
8	115	1,479	7.78	126	1,454	8.67
9	15	198	7.56	16	198	8.07
	**Conditioning on the future approach**					
	Ischemic stroke			Ischemic stroke/systemic embolism		
CHA_2DS_2-VASc score	Events	Person-years	Rate/100 person-years	Events	Person-years	Rate/100 person-years
0	95	32,727	0.29	107	32,712	0.33
1	251	40,415	0.62	261	40,407	0.65
2	756	40,618	1.86	792	40,576	1.95
3	1,678	52,187	3.22	1,766	52,114	3.39
4	1,778	40,213	4.42	1,890	40,107	4.71
5	1,356	22,301	6.08	1,427	22,242	6.42
6	793	10,459	7.58	857	10,392	8.25
7	327	3,751	8.72	349	3,732	9.35
8	68	891	7.63	75	882	8.51
9	10	103	9.71	11	103	10.69
	**Censoring approach**					
	Ischemic stroke			Ischemic stroke/systemic embolism		
CHA_2DS_2-VASc score	Events	Person-years	Rate/100 person-years	Events	Person-years	Rate/100 person-years
0	239	44,382	0.54	261	44,367	0.59
1	521	55,842	0.93	541	55,830	0.97
2	1,247	55,966	2.23	1,305	55,920	2.33
3	2,274	64,838	3.51	2,411	64,750	3.72
4	2,385	48,284	4.94	2,547	48,162	5.29
5	1,772	25,834	6.86	1,900	25,760	7.38
6	1,070	12,087	8.85	1,159	12,012	9.65
7	406	4273	9.50	443	4,253	10.42
8	87	983	8.85	97	973	9.97
9	11	124	8.89	12	124	9.70

eTable 3: Primary diagnosed thromboembolic event rates according to different levels of baseline CHA_2DS_2-VASc score stratified by methodological approach.

CHA_2DS_2-VASc score	Formal rate assessment					
	Ischemic stroke			Ischemic stroke/systemic embolism		
	Events	Person-years	Rate/100 person-years	Events	Person-years	Rate/100 person-years
0	360	75,085	0.48	393	75,004	0.52
1	784	101,932	0.77	823	101,855	0.81
2	1,659	115,033	1.44	1,758	114,870	1.53
3	2,730	124,323	2.20	2,918	124,023	2.35
4	2,691	91,798	2.93	2,885	91,495	3.15
5	1,833	47,998	3.82	1,996	47,726	4.18
6	1,106	22,155	4.99	1,212	21,978	5.51
7	395	7,242	5.45	430	7194	5.98
8	70	1,534	4.56	78	1515	5.15
9	11	204	5.39	11	204	5.39
	**Conditioning on the future approach**					
	Ischemic stroke			Ischemic stroke/systemic embolism		
CHA_2DS_2-VASc score	Events	Person-years	Rate/100 person-years	Events	Person-years	Rate/100 person-years
0	75	32,759	0.23	84	32,748	0.26
1	195	40,509	0.48	204	40,501	0.50
2	576	40,810	1.41	607	40,775	1.49
3	1,306	52,561	2.48	1,385	52,501	2.64
4	1,354	40,723	3.32	1,452	40,629	3.57
5	964	22,862	4.22	1,022	22,810	4.48
6	567	10,772	5.26	622	10,710	5.81
7	234	3,867	6.05	247	3,853	6.41
8	40	929	4.30	44	925	4.76
9	6	109	5.52	6	109	5.52
	**Censoring approach**					
	Ischemic stroke			Ischemic stroke/systemic embolism		
CHA_2DS_2-VASc score	Events	Person-years	Rate/100 person-years	Events	Person-years	Rate/100 person-years
0	204	44,432	0.46	223	44,418	0.50
1	419	55,966	0.75	436	55,957	0.78
2	957	56,196	1.70	1,008	56,157	1.79
3	1,766	65,274	2.71	1,890	65,198	2.90
4	1,792	48,860	3.67	1,937	48,749	3.97
5	1,249	26,432	4.73	1,364	26,366	5.17
6	746	12,435	6.00	823	12,363	6.66
7	288	4,412	6.53	311	4,398	7.07
8	53	1,026	5.17	60	1,021	5.88
9	7	130	5.40	7	130	5.40

eTable 4: Primary diagnosed thromboembolic event rates in relation to different methodological approaches stratified according to cut-off values of stroke risk based on the CHA_2DS_2-VASc score.

Risk stratification	CHA_2DS_2-VASc score	Formal rate assessment			Conditioning on the future approach			Censoring at oral anticoagulant treatment		
		Events	Person-years	Rate/100 person-years	Events	Person-years	Rate/100 person-years	Events	Person-years	Rate/100 person-years
Truly low risk	0 (1 for females)	566	114,771	0.49	131	56,121	0.23	331	73,964	0.45
European treatment threshold	1 (males)	650	62,088	1.05	157	17,129	0.92	328	26,412	1.24
U.S. treatment threshold	2	1,758	114,870	1.53	607	40,775	1.49	1,008	56,157	1.79
High risk	>2	9,530	294,135	3.24	4778	131,537	3.63	6,392	158,225	4.04

eTable 5: Thromboembolic event rates according to different levels of baseline CHA_2DS_2-VASc score using data from previous five years (2009 – 2014).

	Formal rate assessment, N=74,462					
	Ischemic stroke			Ischemic stroke/systemic embolism		
CHA_2DS_2-VASc score	Events	Person-years	Rate/100 person-years	Events	Person-years	Rate/100 person-years
0	103	18,382	0.56	111	18,371	0.60
1	237	26,202	0.90	253	26,174	0.97
2	508	29,919	1.70	535	29,896	1.79
3	855	31,819	2.69	905	31,763	2.85
4	965	25,478	3.79	1,024	25,407	4.03
5	787	13,685	5.75	828	13,640	6.07
6	534	6,692	7.98	565	6,647	8.50
7	182	2,320	7.84	195	2,305	8.46
8	37	609	6.07	40	609	6.57
9	3	78	3.84	3	78	3.84
